# Targeted Delivery of Chemo‐Sonodynamic Therapy via Brain Targeting, Glutathione‐Consumable Polymeric Nanoparticles for Effective Brain Cancer Treatment

**DOI:** 10.1002/advs.202203894

**Published:** 2022-08-15

**Authors:** Haoan Wu, Xingchun Gao, Yuanyuan Luo, Jiang Yu, Gretchen Long, Zhaozhong Jiang, Jiangbing Zhou

**Affiliations:** ^1^ Department of Neurosurgery Yale University New Haven CT 06510 USA; ^2^ Department of Biomedical Engineering Yale University New Haven CT 06510 USA; ^3^ Integrated Science and Technology Center Yale University 600 West Campus Drive West Haven CT 06516 USA

**Keywords:** blood–brain barrier, brain tumor, chemo‐sonodynamic, GSH, shrinkable nanoparticles

## Abstract

Glioblastoma (GBM) is the most aggressive tumor of the central nervous system and remains universally lethal due to lack of effective treatment options and their inefficient delivery to the brain. Here the development of multifunctional polymeric nanoparticles (NPs) for effective treatment of GBM is reported. The NPs are synthesized using a novel glutathione (GSH)‐reactive poly (2,2″‐thiodiethylene 3,3″‐dithiodipropionate) (PTD) polymer and engineered for brain penetration through neutrophil elastase‐triggered shrinkability, iRGD‐mediated targeted delivery, and lexiscan‐induced autocatalysis. It is found that the resulting lexiscan‐loaded, iRGD‐conjugated, shrinkable PTD NPs, or LiPTD NPs, efficiently penetrate brain tumors with high specificity after intravenous administration. Furthermore, it is demonstrated that LiPTD NPs are capable of efficient encapsulation and delivery of chemotherapy doxorubicin and sonosensitizer chlorin e6 to achieve combined chemotherapy and sonodynamic therapy (SDT). It is demonstrated that the capability of GSH depletion of LiPTD NPs further augments the tumor cell killing effect triggered by SDT. As a result, treatment with LiPTD NPs effectively inhibits tumor growth and prolongs the survival of tumor‐bearing mice. This study may suggest a potential new approach for effective GBM treatment.

## Graphical abstract

Glutathione (GSH)‐reactive polymer‐based nanoparticles (NPs), which can target drug delivery to the brain tumor through the integration of neutrophil elastase‐triggered shrinkability, ligand‐mediated interaction, and lexiscan‐induced blood–brain barrier modulation. The resulting NPs with excellent penetration capability can efficiently deliver chemotherapy drug doxorubicin and sonosensitizer chlorin e6 to tumors in the brain for effective chemo‐sonodynamic combination therapy.

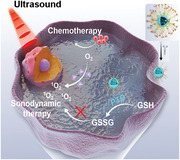

## 1 Introduction

Glioblastoma multiforme (GBM, grade IV astrocytoma) is the most common and aggressive malignant glioma, with a median survival rate of less than 16 months after diagnosis.^[^ ^1^ ^]^ The current standard of care has remained unchanged for over a decade, and involves maximal surgical resection followed by chemotherapy and radiotherapy. Despite extensive research efforts, current therapeutics for GBM remain ineffective. This can be attributed to the blood–brain barrier (BBB) limiting therapeutic entry, and therapeutic resistance.^[^ ^2^ ^]^ Therefore, novel approaches that simultaneously address these challenges are greatly needed to improve GBM treatment and outcome.^[^ ^3^ ^]^

Nanotechnology is a promising avenue for delivering therapeutics to GBM tumor sites, since nanoparticles (NPs) can be engineered for crossing the BBB.^[^ ^4^, ^5^ ^]^ The blood–brain barrier (BBB), comprised of tight junctions between endothelial cells, limits drug delivery to the brain.^[^ ^4^, ^5^, ^6^ ^]^ Although the BBB is partially compromised in brain tumors, the amount of pharmacological agents delivered into the brain remains inadequate for effective GBM treatment.^[^ ^4^, ^5^ ^]^ Currently, NPs have begun to address this challenge through surface conjugation of molecules that recognize molecules highly expressed on the BBB, such as transferrin receptors (TfR), or those enriched in the GBM tumor microenvironment (TME), such as matrix metalloproteinase‐2 (MMP‐2).^[^ ^7^, ^8^, ^9^ ^]^ Despite some promise, the ligand‐mediated approach alone is often insufficient for effective GBM treatment.^[^ ^10^, ^11^, ^12^ ^]^ To further improve the efficiency, several new approaches were recently explored. One approach includes engineering NPs to respond to the TME by altering their physical properties, such as shrinking or expanding in size, complementary to the ligand‐mediated targeting approach to increase tumor targeting efficiency.^[^ ^13^, ^14^, ^15^, ^16^, ^17^ ^]^ In addition to direct chemical modification, encapsulation of BBB modulatory molecules, such as lexiscan, has also shown to significantly augment the delivery efficiency of the ligand‐mediated approach through an autocatalytic mechanism.^[^ ^17^, ^18^, ^19^ ^]^ Using this method, a small amount of NPs penetrate the brain after the first administration and release BBB modulators into brain tissue to locoregionally open the BBB, leading to enhanced delivery efficiency in subsequent administrations.

Accumulating evidence suggests that GBM tumors are highly resistant to conventional chemotherapy.^[^ ^20^, ^21^, ^22^ ^]^ To overcome this drug resistance, a combination of conventional chemotherapy with various treatment modalities, such as photodynamic therapy, has been explored.^[^ ^23^, ^24^ ^]^ Sonodynamic therapy (SDT) employs ultrasound (US) and sonosensitizers to trigger the generation of singlet oxygen (^1^O_2_) with deeper penetration into the body and is shown to have the potential to reduce drug tolerance as a reactive oxygen species (ROS)‐based anticancer approach.^[^ ^25^, ^26^, ^27^ ^]^ However, the therapeutic benefit of SDT for cancer treatment is often limited, because the TME contains a high concentration of glutathione (GSH), which consumes the as‐produced ^1^O_2_.^[^ ^28^, ^29^ ^]^ Therefore, in this study, we developed and evaluated multifunctional NPs consisting of GSH‐reactive poly (2,2″‐thiodiethylene 3,3″‐dithiodipropionate) (PTD) polymers for targeted delivery of chemo‐sonodynamic therapy to GBM. The PTD NPs were designed to have two main functions. In addition to serving as a drug carrier, the NPs function as an endogenous GSH depletion agent. The NPs were functionalized for brain penetration through neutrophil elastase (NE)‐triggered shrinkability, iRGD‐mediated targeted delivery and lexiscan‐induced autocatalysis. We found that the resulting lexiscan‐loaded, iRGD‐conjugated, shrinkable PTD NPs, or LiPTD NPs, efficiently penetrated tumors in the brain, and when encapsulated with the chemotherapy drug doxorubicin (DOX) and sonosensitizer chlorin e6 (Ce6), the NPs enabled effective chemo‐sonodynamic combination therapy.

## 2 Results

**Figure 1:**
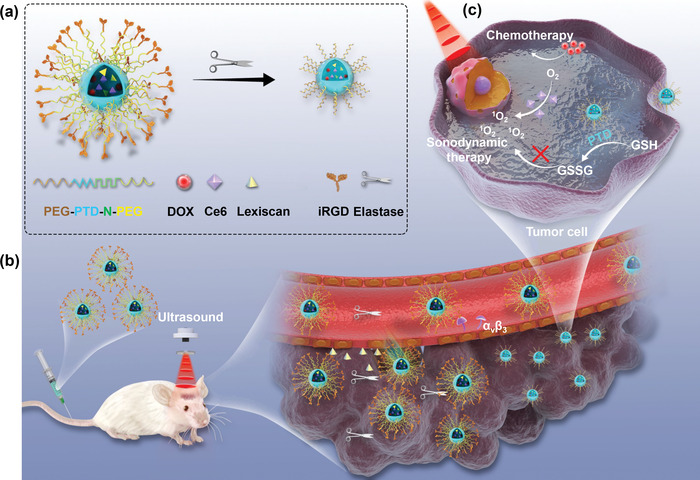
Schematic diagrams of LiPTD NPs design and combination therapy for GBM treatment. a) Construction of LiPTD NPs through self‐assembly of PEG‐PTD‐N‐PEG block copolymer and size shrink behavior of LiPTD NPs in response to NE. b) Brain targeting and BBB penetration of LiPTD NPs in tumor‐bearing mice. c) Chemo‐sonodynamic therapy for tumor cells under US irradiation.

Effective treatment of GBM requires efficient delivery of multimodal therapies to tumors in the brain. In this study, we designed and evaluated multifunctional NPs for GBM treatment through the delivery of chemo‐sonodynamic combination therapy. Evidence suggests that the elevated level of GSH in the TME represents a major cause of inadequate effect in ROS‐based treatment.^[^ ^30^, ^31^ ^]^ To eliminate endogenous GSH, we synthesized poly(ethylene glycol) (PEG)‐terminated PTD (PEG‐PTD) polymers, which contain GSH‐reactive disulfide groups. To achieve intelligent responsiveness, PEG‐PTD polymers were conjugated with a second PEG through a neutrophil elastase (NE)‐cleavable peptide, NH_2_‐RLQLKL‐C‐SH.^[^ ^32^ ^]^ The resulting PEG‐flanked polymers can self‐assemble into micellular NPs that are responsive to NE, a protease highly enriched in the GBM tumor microenvironment (Figure S1, Supporting Information), by shrinking in size. To enable tumor targeting, the NPs were conjugated with iRGD on the surface, which recognizes *α*_v_ *β*_3_, integrin highly expressed on tumor vessels and cells. To further enhance the brain penetrability, lexiscan, a BBB modulator that can biologically open the BBB,^[^ ^17^, ^18^, ^19^ ^]^ was encapsulated in NPs. The resulting LiPTD NPs were characterized in tumor‐bearing mice as a carrier for targeted delivery of DOX, a chemotherapy drug, and Ce6, a SDT sonosensitizer, to the brain for tumor treatment (**Figure** **1**).

### 2.1 Synthesis and Characterization of NE‐Responsive Polymers

**Figure 2:**
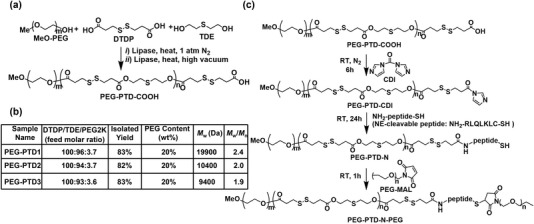
Synthesis and characterization of polymers. a) Enzyme‐mediated preparation of carboxyl‐terminated PEG‐PTD block copolymer. b) Characterization for a family of three PEG‐PTD block copolymers. c) Chemical synthesis route of PEG‐PTD‐N and PEG‐PTD‐N‐PEG.

Using Novozym 435 catalyst (N435), an enzyme highly tolerant of organic functional groups including tertiary amino, thioether and disulfide moieties,^[^ ^33^, ^34^, ^35^ ^]^ a group of PEG‐PTD block copolymers were synthesized through copolymerization of 3,3″‐dithiodipropionic acid (DTDP), 2,2″‐thiodiethanol (TDE) and MeO‐PEG2K‐OH (**Figure** **2**a). The use of excessive DTDP versus (TDE + PEG) effectively controls the polymer molecular weight and ensures that the polymer chain termini are predominantly carboxyl groups. In this reaction, all copolymers with 20 wt% PEG content were formed by adding calculated amounts of MeO‐PEG2K‐OH. Three PEG‐PTD copolymers, termed as PEG‐PTD1, PEG‐PTD2, and PEG‐PTD3, were obtained in a yield of 82–83% with molecular weights of 19.900, 10.400, and 9.400 Da, respectively (Figure 2b).

The structures of PEG‐PTD copolymers were analyzed and confirmed using ^1^H and ^13^C NMR spectroscopy. The structural assignments of polymers for the proton and carbon‐13 resonance absorptions are described in Figure S2, Supporting Information. To enable the responsiveness of NE, a second PEG block was further conjugated to the end of the polymers via a NE‐cleavable peptide linker, NH_2_‐RLQLKL‐C‐SH (Figure 2c). Briefly, carboxyl‐terminated PEG‐PTD polymer was preactivated by *N*, *N*′‐carbonyl diimidazole (CDI) and then reacted with NH_2_ on the peptide for 24 h at room temperature, resulting in the PEG‐PTD‐peptide (PEG‐PTD‐N) with a cysteine residue. Next, maleimide‐terminated PEG5k (PEG in a molecular weight of 5 kDa) was mixed with PEG‐PTD‐N at room temperature for 1 h to obtain resulting block copolymers bearing NE‐cleavable peptide, designated as PEG‐PTD‐N‐PEG. Control nonresponsive polymers, PEG‐PTD‐PEG, were synthesized by the reaction of CDI preactivated PEG‐PTD and amine‐terminated PEG5k (MeO‐PEG5K‐NH_2_) directly without the peptide.

### 2.2 Synthesis and Evaluation of PEG‐PTD‐N‐PEG NPs for Targeted Drug Delivery to Brain Tumors

**Figure 3:**
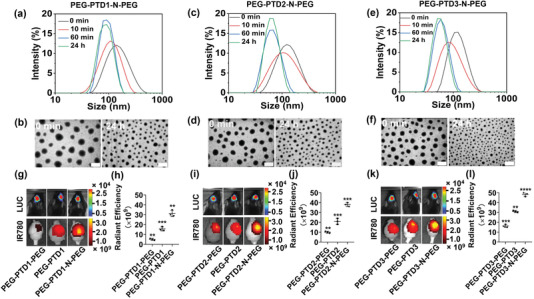
Characterization and investigation of NE responsive PEG‐PTD‐N‐PEG NPs. a–f) DLS and TEM analyses of PEG‐PTD‐N‐PEG NPs in PBS with NE (100 nm). PEG‐PTD1‐N‐PEG NPs a,b), PEG‐PTD2‐N‐PEG NPs c,d), PEG‐PTD3‐N‐PEG NPs e,f). Scale bar: 100 nm. g–l) Representative images g,i,k) and semiquantification h,j,l) of NPs in tumor bearing mice receiving the indicated treatments.

PEG‐PTD‐N‐PEG copolymers were designed for the formation of NPs that are responsive to NE by shrinking in size.^[^ ^17^ ^]^ Mechanistically, PEG‐PTD‐N‐PEG micellular NPs consist of two layers of PEG on the surface. PEG molecules conjugated via the NE‐responsive peptide were cleaved after exposure to NE. Consequently, the balance of hydrophobicity and hydrophilicity was rearranged, resulting in a new micellular structure, which contains a higher percentage of hydrophobic components compared to the original NPs. The high hydrophobic content facilitates shrinkage of NP size. To validate the design, PEG‐PTD‐N‐PEG NPs were fabricated through standard nanoprecipitation procedures (see the Experimental Section, Construction of NPs) and incubated with 100 nm NE at 37 °C. The change of size and morphology of NPs with time were determined. Dynamic light scattering (DLS) analysis showed that the hydrodynamic size of all three NPs started to shrink 10 min after incubation with NE. After 24 h of incubation, the size shrank from 145 to 88 nm, 132 to 68 nm, and 121 to 54 nm, for NPs consisting of PEG‐PTD1‐N‐PEG, PEG‐PTD2‐N‐PEG, and PEG‐PTD3‐N‐PEG, respectively. The changes in size were confirmed by transmission electron microscopy (TEM), and the morphology of the NPs remained spherical (**Figure** **3**a–f). In contrast, control nonresponsive NPs, which were prepared using PEG‐PTD‐PEG or PEG‐PTD, did not show size alterations (Figures S3 and S4, Supporting Information). We found that without NE the NPs were stable in media at different pHs over 30 days, confirming that the observed changes in size were induced by NE (Figure S5, Supporting Information). In addition, all the NPs appear to be safe and did not exhibit significant cytotoxicity or hemolytic activity at a concentration up to 500 µg mL^−1^ (Figure S6, Supporting Information).

We evaluated if NE‐mediated shrinkability enhances the delivery of PEG‐PTD‐N‐PEG NPs to brain tumors. NPs were synthesized with the encapsulation of IR780, a near‐infrared fluorescence dye that allows for noninvasive imaging. The resulting NPs were intravenously administered to mice 3 weeks after inoculation of luciferase‐expressing GL261 cells, the most widely used syngeneic model cells of murine glioblastoma.^[^ ^36^ ^]^ Twenty‐four hours later, the mice were imaged using an in vivo imaging system (IVIS). Afterward, the mice were euthanized and the brains were isolated and imaged ex vivo. We found that, while all experimental mice had tumors of a comparable size, compared to control nonresponsive PEG‐PTD‐PEG or PEG‐PTD NPs, all three PEG‐PTD‐N‐PEG NPs demonstrated significantly greater efficiency in brain tumor penetration. Quantification based on fluorescence intensity showed that the accumulation of PEG‐PTD1‐N‐PEG shrinkable NPs in the tumor region was 2.1‐ and 6.2‐fold greater than those of PEG‐PTD1 and PEG‐PTD1‐PEG NPs, respectively (Figure 3g,h). A similar trend of enhancement was found for PEG‐PTD2‐N‐PEG and PEG‐PTD3‐N‐PEG NPs (Figure 3i–l). Among all the three NE‐responsive NPs, PEG‐PTD3‐N‐PEG NPs demonstrated the greatest efficiency and, thus, were selected for the remainder of the study. Gel permeation chromatography (GPC) analysis confirmed that polymers were cleaved and had reduced molecular weight after shrinking (Figure S7, Supporting Information). We found that, while both have a comparable size after NE treatment in vitro (Figure 3; and Figure S4, Supporting Information), PEG‐PTD‐N‐PEG NPs had an efficiency greater than nonresponsive PEG‐PTD NPs in brain tumor penetration (Figure 3g–l). To further understand the potential mechanism related to NE, we compared the accumulation of PEG‐PTD3‐N‐PEG NPs with and without coadministration of BAY‐678, a NE inhibitor. We found that treatment with BAY‐678 effectively inhibited the tumor‐penetration ability of PEG‐PTD3‐N‐PEG NPs to a level comparable to that of PEG‐PTD3 (Figure S8, Supporting Information). This finding, which is consistent with previous reports,^[^ ^15^, ^16^, ^17^ ^]^ suggests stimuli‐triggered shrinkability as a mechanism to enhance targeted delivery of NPs to disease microenvironment.

### 2.3 Characterization of PEG‐PTD‐N‐PEG NPs for Delivery of SDT

SDT kills cells through the generation of oxidation products.^[^ ^25^, ^26^, ^27^ ^]^ Unfortunately, the therapeutic potential of SDT for cancer treatment is limited by the highly reducing TME, which contains a high level of GSH and can consume the oxidation products induced by SDT.^[^ ^37^, ^38^ ^]^ Effective SDT for cancer treatment requires decreasing GSH in the TME.

**Figure 4:**
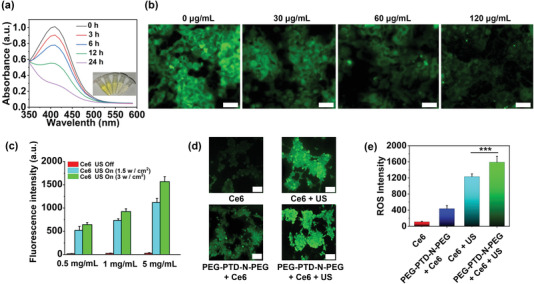
Characterization of PEG‐PTD‐N‐PEG NPs for GSH depletion and ROS generation. a) Time‐dependent GSH depletion by PEG‐PTD‐N‐PEG NPs. The inset in (a) is a photograph of the solutions. b) GSH detection images of GL261 cells stained with Thiol Tracker violet after incubation with various concentration of PEG‐PTD‐N‐PEG NPs. Scale bar: 50 µm. c) Singlet oxygen detection of indicated groups with various concentration of PEG‐PTD‐N‐PEG NPs. d) Representative images and e) semiquantification of ROS levels in GL261cells stained with DCFH‐DA after various treatments. Scale bar: 100 µm.

PEG‐PTD‐N‐PEG polymers contain disulfide groups, which react with GSH,^[^ ^39^, ^40^ ^]^ and have the potential to function as GSH depletion agents to improve SDT efficacy. To test the hypothesis, we determined the ability of PEG‐PTD‐N‐PEG NPs to react with and deplete GSH, which was quantified using 5, 5'‐dithiobis‐(2‐nitrobenzoic acid) (DTNB). We found that PEG‐PTD‐N‐PEG NPs reacted with GSH and consumed up to 81% of GSH after 24 h incubation (**Figure** **4**a). We further evaluated if treatment with PEG‐PTD‐N‐PEG NPs could reduce GSH within cancer cells. Thiol Tracker staining analysis showed that treatment with the NPs efficiently reduced intracellular GSH in a dose‐dependent manner (Figure 4b).

Lastly, we evaluated PEG‐PTD‐N‐PEG NPs for SDT delivery. Ce6‐loaded PEG‐PTD‐N‐PEG NPs were synthesized with encapsulation of Ce6 at 3.1% by weight. To investigate, if US promotes ROS generation by Ce6, we employed singlet oxygen sensor green (SOSG) as a probe to monitor ^1^O_2_ generation. We found that the ^1^O_2_ generation capacity of Ce6‐loaded PEG‐PTD‐N‐PEG NPs was positively correlated with both the strength of US irradiation and the concentration of NPs (Figure 4c). To determine if the NPs can generate ROS within cells, we treated GL261 cells with Ce6‐loaded PEG‐PTD‐N‐PEG NPs and monitored the change of intracellular ROS using 2,7‐dichlorofluorescein diacetate (DCFH‐DA) after application of US. We found that, while all treatments generated ROS within cells, delivery via PEG‐PTD‐N‐PEG NPs enhanced the ROS production efficiency of Ce6 (Figure 4d,e). This is likely due to PEG‐PTD‐N‐PEG NPs consumption of intracellular GSH (Figure 4a,b), and thus stabilized ROS.

### 2.4 Engineering PEG‐PTD‐N‐PEG NPs for Targeted Delivery to Brain Tumors

**Figure 5:**
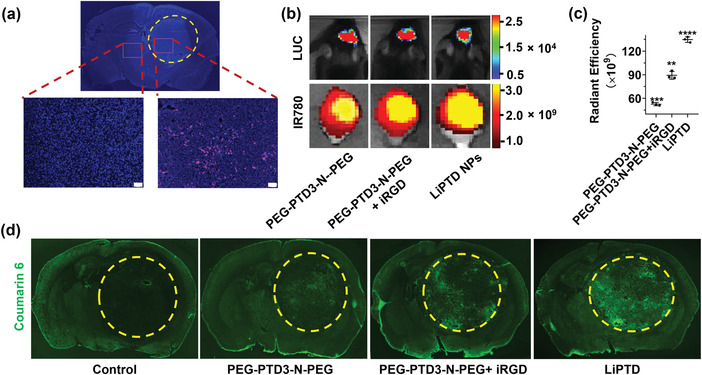
Enhancing delivery of PEG‐PTD‐N‐PEG NPs to gliomas in vivo. a) Immunostaining images of the expression of *α*_v_ *β*_3_ in the region with (yellow dotted circle) and without tumor. Scale bar: 100 µm. b) Representative images, and c) semiquantification of NPs in tumor bearing mice received the indicated treatments. d) Representative fluorescence images of Coumarin 6‐loaded NPs (green) in the tumor regions (yellow dotted circle).

The BBB in brain tumors is partially compromised,^[^ ^4^, ^5^ ^]^ allowing a fraction of PEG‐PTD‐N‐PEG NPs to accumulate in tumors after intravenous administration (Figure 3). To further improve the delivery efficiency and specificity, we focused on *α*_v_ *β*_3_, an integrin that is expressed in tumor neovasculature and can be efficiently targeted through peptide iRGD.^[^ ^41^ ^]^ Analysis by immunostaining confirmed that *α*_v_ *β*_3_ was highly expressed in the tumor region but not in normal brain tissues (**Figure** **5**a). To enable convenient surface modification, we synthesized carboxyl‐terminated PEG‐PTD‐N‐PEG through the same procedures that were developed for the synthesis of PEG‐PTD‐N‐PEG, except the use of MAL‐PEG‐COOH to replace the MeO‐PEG‐MAL. To enable conjugation, iRGD with amine was reacted with carboxyl groups displayed on the surface of PEG‐PTD‐N‐PEG NPs. We evaluated PEG‐PTD‐N‐PEG NPs with and without iRGD in mice bearing GL261 tumors. Results in Figure 5b,c showed that surface conjugation of iRGD enhanced the delivery of NPs to tumors by 1.7‐fold.

Recently, we designed and validated autocatalysis, which can be achieved through encapsulation of BBB modulators, such as lexiscan, as a novel approach to improve the delivery efficiency of the ligand‐mediated delivery approach.^[^ ^42^, ^43^ ^]^ To test if autocatalysis works for PEG‐PTD‐N‐PEG NPs, we synthesized lexiscan‐loaded (1% by weight), iRGD‐conjugated, PEG‐PTD‐N‐PEG NPs, or LiPTD NPs, with and without encapsulation of IR780 and evaluated them in mice bearing GL261 gliomas. To achieve autocatalysis in vivo, tumor bearing mice were primed with LiPTD NPs without IR780 daily for two consecutive days and treated with IR780‐loaded LiPTD NPs at day 3. Twenty‐four hours after the last treatment, the mice were imaged. Afterward, the mice were euthanized, and major organs were isolated and imaged ex vivo. We found that priming with lexiscan‐loaded NPs enhanced delivery of PEG‐PTD‐N‐PEG NPs by 1.6‐fold (Figure 5b,c). This finding was further validated by fluorescence‐based microscope imaging, in which tumor bearing mice were treated with LiPTD NPs encapsulating coumarin 6, a fluorescence dye (Figure 5d). Of note, ex vivo imaging of isolated organs showed that the amount of LiPTD NPs in the brain is greater than that in the liver, which has the greatest NP accumulation among all peripheral organs (Figure S9, Supporting Information). Evans‐blue leakage assay confirmed that priming with lexiscan‐loaded NPs significantly increased BBB permeability selectively in tumors (Figure S10 Supporting Information). The excellent penetrability of LiPTD NPs was confirmed in an in vitro BBB model, which was established by growing astrocytes and endothelial cells on the basolateral and apical side of the membrane, respectively.^[^ ^44^, ^45^ ^]^ We found that LiPTD NPs crossed the artificial BBB in a time‐dependent manner, while PEG‐PTD3‐PEG NPs had significantly less penetrability (Figure S11, Supporting Information). We found that LiPTD NPs had excellent serum stability and long blood circulation time after intravenous administration (Figure S12, Supporting Information). Cellular uptake analysis showed that LiPTD NPs could be rapidly internalized by GL261 cells (Figure S13, Supporting Information). Due to their high efficiency in targeted delivery to brain tumors and excellent serum stability and blood circulation, LiPTD NPs were selected for the rest of the study.

### 2.5 Targeted Delivery of Chemo‐Sonodynamic Combination Therapy via LiPTD NPs for GBM Treatment

**Figure 6:**
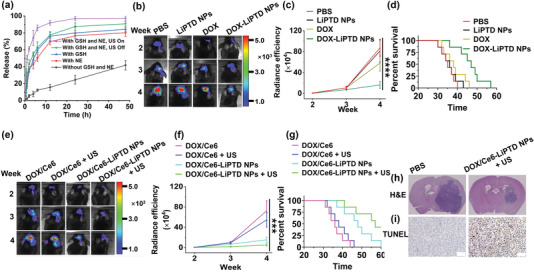
Evaluation of DOX/Ce6‐loaded LiPTD NPs for GBM treatment. a) Release of DOX in PBS at 37 °C under the indicated conditions. b) Representative images and c) semiquantification of luciferase signals in brain tumors in mice received the indicated treatments. d) Kaplan–Meier survival analysis of mice received the indicated treatments (*n* = 7). e) Representative images and f) semiquantification of luciferase signals in brain tumors in mice received the indicated treatments. g) Kaplan–Meier survival analysis of mice received the indicated treatments (*n* = 7). h,i) Representative immunohistochemical staining of brain tumors isolated from mice received the indicated treatments. Scale bar: 100 µm.

Although it is one of the most frequently used chemotherapy drugs, DOX is not utilized for the clinical management of GBM due to its limited ability to penetrate the brain.^[^ ^46^ ^]^ We therefore assessed LiPTD NPs for systemic delivery of DOX and Ce6 to the brain for GBM treatment. LiPTD NPs were synthesized with coencapsulation of DOX and Ce6. The resulting DOX/Ce6‐loaded LiPTD NPs, or DOX/Ce6‐LiPTD NPs, contained DOX and Ce6 at 4.2% and 3.4% by weight, respectively. As expected, LiPTD NPs released encapsulated DOX at an accelerated rate in the presence of both GSH and NE. Twenty‐four hours after incubation in PBS with GSH and NE, 87.6% of DOX was released; in contrast, only 24.5% was released in PBS without GSH and NE at the same condition (**Figure** **6**a). Application of US irradiation further promoted drug release. The accumulative release of DOX increased to 97.3% at 24 h after 3 W cm^−2^ ultrasonication (3 MHz, 3 min). This accelerated drug release could be attributed to ROS generated by Ce6 after US irradiation, which oxidizes the thioether units within PTD polymers, leading to swelling of NPs and acceleration of drug release (Figure S14, Supporting Information).

We assessed DOX/Ce6‐LiPTD NPs for systemic treatment of GBMs. First, we evaluated LiPTD NPs for delivery of chemotherapy. Mice bearing GL261 gliomas were established, randomly grouped and treated with PBS, or DOX (2 mg kg^−1^), or blank LiPTD NPs, or DOX‐LiPTD NPs (dose equivalent to 2 mg DOX kg^−1^). Treatments were started 1 week after tumor inoculation and performed twice a week for three consecutive weeks. The mice were monitored for tumor growth by luciferase imaging, body weight change, and survival. IVIS analysis showed that free DOX had a moderate tumor inhibition effect, while its efficacy was significantly enhanced when delivered via LiPTD NPs (Figure 6b,c). Next, we evaluated if the therapeutic benefit of DOX‐LiPTDs could be further enhanced through combination with codelivery of sonodynamic therapy. A separate cohort of tumor bearing mice were prepared and treated with DOX/Ce6‐LiPTD NPs with US, or DOX/Ce6‐LiPTD NPs + US. NPs were given at a dose equivalent to 2 mg DOX and 1.6 mg Ce6 kg^−1^. Control mice received treatment of the same amount of DOX/Ce6, or DOX/Ce6 + US, or DOX/Ce6‐LiPTD NPs without US. Treatments were performed according to the same procedures with chemotherapy except the US irradiation (3 MHz, 3 W cm^−2^, 10 min) was applied 24 h after intravenous administration. IVIS imaging showed that treatments with DOX/Ce6 and DOX/Ce6 + US showed similar tumor inhibition effects with DOX. Both of them were less than that of treatment with DOX/Ce6‐LiPTD NPs, and the tumor inhibition effect of DOX/Ce6‐LiPTD NPs were significantly improved when US was applied (Figure 6e,f). Consistently, Kaplan–Meier survival analysis found that treatment with DOX/Ce6‐LiPTD NPs + US significantly enhanced the survival of GBMs‐bearing mice (*p* < 0.05). The median survival in the group receiving treatment of DOX/Ce6‐LiPTD NPs + US was 57 days, compared to 35, 36, 37, 37, 38, 47, and 48 days for the group treated with PBS, LiPTD NPs, DOX, DOX/Ce6, DOX/Ce6 + US, DOX‐LiPTD NPs, and DOX/Ce6‐LiPTD NPs, respectively (Figure 6d,g). A separate cohort of mice was euthanized 28 days after inoculation. The brains were harvested and subjected to pathological analysis. H&E staining showed that, compared to control PBS group, the group treated with DOX/Ce6‐LiPTD NPs + US had significantly smaller tumor lesions (Figure 6h; and Figure S15a, Supporting Information). Terminal deoxynucleotidyl transferase dUTP nick end labeling (TUNEL) staining found that treatment with DOX/Ce6‐LiPTD NPs + US induced marked cellular apoptosis to a degree that was significantly greater than those in other treatment groups (Figure 6i; and Figure S15b, Supporting Information). GSH staining assay confirmed that treatment with LiPTD NPs significantly reduced the level of GSH in tumors (Figure S16, Supporting Information).

To enhance validity of the findings, we characterized LiPTD NP‐based chemo‐sonodynamic therapy in a second mouse model that was established through inoculation of human U87 glioma cells. The experiments were performed according to the same procedures described above. Consistently, we found that delivery via LiPTD NPs significantly improved the efficacy of DOX‐mediated chemotherapy and codelivery of Ce6‐mediated sonodynamic therapy resulted in therapeutic benefits greater than those by all control treatments (Figure S17, Supporting Information).

Treatment with DOX/Ce6‐LiPTD NPs appeared to be safe. Throughout the study, no significant loss of body weight was found in all the treatment groups (Figure S18, Supporting Information). H&E staining showed that DOX/Ce6‐LiPTD NPs + US did not induce detectable toxicity or damage to major organs (Figure S19, Supporting Information). Alanine aminotransferase (ALT) assay and aspartate aminotransferase (AST) assay did not identify significant hepatotoxicity after intravenous administration of the LiPTD NPs (Figure S20, Supporting Information).

## 3 Discussion

Effective treatment of GBM requires the development of multimodal therapeutics that can also be efficiently delivered to tumors in the brain.^[^ ^20^, ^21^, ^22^ ^]^ In this study, we synthesized DOX/Ce6‐loaded LiPTD NPs to simultaneously address both challenges. The NPs consisted of optimized PTD polymers that were synthesized with encapsulation of both the chemotherapy drug DOX and sonosensitizer Ce6. Upon US irradiation, Ce6 generates ROS within cells, which kills tumor cells and sensitizes tumor cells to chemotherapy. Unlike traditional polymers, such as poly(lactic‐*co*‐glycolic acid), which acts as a drug carrier without biological functions, PTD contains disulfide groups that can react with GSH and thus can improve the anti‐cancer activities of ROS‐based SDT. The dramatically accelerated DOX release from the NPs in TME under the optimal treatment conditions ensures maximal potency of the chemotherapeutic drug in killing cancer cells (Figure 6a). The NPs were further engineered for penetrating tumors in the brain through three complementary mechanisms. First, through the incorporation of a protease‐responsive peptide into the polymer chain, the NPs were engineered to respond to NE enriched in the TME by shrinking in size (Figure 3). Although the concentration of NE in the blood was elevated to 3.9 nm 3 weeks after tumor inoculation (Figure S21a, Supporting Information), it remains below the concentration that is required for cleaving the NE‐responsive peptide (Figure 3a–f; and Figure S21b, Supporting Information). Therefore, LiPTD NPs were able to maintain their structure in blood circulation before reaching tumors in the brain. Second, the NPs were surface conjugated with iRGD, which mediated targeted delivery to tumors through interaction with *α*_v_ *β*_3_ preferentially expressing on the tumor vasculature (Figure 5a). Third, the NPs were encapsulated with lexiscan to achieve autocatalysis (Figure 5b,c). As a result, LiPTD NPs after intravenous administration accumulated in tumors in the brain with high efficiency (Figure 5d). We showed that, when DOX and Ce6 were coencapsulated and delivered, LiPTD NPs effectively inhibited tumor growth and prolonged survival of tumor‐bearing mice with limited systemic toxicity; in contrast, delivery of a dose‐equivalent amount of free drug did not exhibit comparable efficacy (Figure 6; and Figure S17, Supporting Information).

Although ultrasound has a great ability to penetrate soft tissue, its strength could be significantly attenuated by the skull.^[^ ^47^ ^]^ In this study, we found that application of ultrasound was sufficient to generate effective sonodynamic effect, which could be potentially attributed to three reasons. First, all the sonodynamic studies were performed in mouse models of gliomas. To establish these mouse models, a burr hole was physically made on the skull. When applying ultrasound, the transducer was placed directly above the burr hole, which allows penetration of ultrasound into brain tissue. In clinical practice, although making burr holes is impractically, a similar technique, thinned‐skull surgery, is often employed.^[^ ^48^, ^49^ ^]^ Second, across all the ultrasound studies, coupling gel was applied between the transducer and the skull to prevent reduction of ultrasonic waves in the air; Third, Ce6‐mediated sonodynamic therapy is highly sensitive and does not require a significant amount of ultrasound energy.^[^ ^50^, ^51^ ^]^ To provide direct evidence that application of ultrasound is sufficient for generation of sonodynamic therapy in the mouse models, we injected 5 µL of Ce6‐loaded LiPTD NPs (Ce6/polymer = 3%, w/w) together with SOSG reagent (20 µm, fluorescent probe for ^1^O_2_) into tumors in the brain and measured the production of singlet oxygen ^1^O_2_ after application of ultrasound by quantifying the fluorescence intensity in tumor tissues (Figure S22, Supporting Information). We found that application of ultrasound effectively increased ^1^O_2_ production by 12 times, suggesting that ultrasound reached tumors and induced significant sonodynamic effects.

## 4 Conclusion

In summary, using novel disulfide‐containing polymers, we developed LiPTD NPs, which enable efficient drug delivery to brain tumors through ligand‐targeted interaction, shrinkability, and autocatalysis mechanisms, and enhanced ROS‐based cancer therapy by consuming GSH. We showed that, when DOX and Ce6 were codelivered, the NPs resulted in combined chemo‐sonodynamic therapy, leading to effective treatment of GBM. Due to their high efficiency in penetrating tumors in the brain and their inherent biological activities, LiPTD NPs have the potential to be employed as a new platform for delivery of multimodal therapies for GBM treatment.

## 5 Experimental Section

### PEG‐PTD‐COOH Synthesis

DTDP, TDE, and MeO‐PEG2K‐OH were copolymerized in diphenyl ether (200 wt% vs total substrate) using 10 wt% Novozym 435 catalyst. The polymerization reactions were carried out at 90 °C initially under one atmospheric pressure of nitrogen gas for 23 h, and subsequently under 1.6 mmHg vacuum for 3 days. The formed polymer products were precipitated in n‐hexane, washed with n‐hexane twice, and then dissolved in chloroform. After filtration to remove the catalyst particles, the filtrates were concentrated under vacuum and reprecipitated in n‐hexane. The final purified copolymers were dried at 30 °C overnight under high vacuum (< 1.0 mmHg). The molecular weight of polymers was measured using GPC, which was calibrated with narrow polydispersity polystyrene standards.

### Synthesis of PEG‐PTD‐N‐PEG

The carboxyl group on PEG‐PTD polymer was activated by CDI. In a typical procedure, PEG‐PTD‐COOH (0.01 mmol) and CDI (0.1 mmol) were dissolved in dichloromethane and stirred overnight at room temperature. PEG‐PTD‐CDI was precipitated by adding excess cold ethanol to above solution. The resulting product was dried under vacuum and stored at 4 °C. Then, PEG‐PTD‐CDI (0.002 mmol) and NH_2_‐RLQLKL‐C‐SH peptide (0.004 mmol) were codissolved in anhydrous DMSO and stirred overnight at room temperature. After that, MeO‐PEG5K‐MAL/MAL‐PEG5K‐COOH (0.005 mmol) can be conjugated to PEG‐PTD‐peptide through mixing them for 1 h at room temperature.

### Construction of NPs

NPs were constructed using standard nanoprecipitation methods. DMSO solution of PEG‐PTD‐N‐PEG polymer was dropwise added to deionized water (DMSO/water = 1:10) under magnetic stirring. Then the solution was stirred for 30 min and dialyzed against deionized water with a dialysis bag (100 000 MWCO). The NPs were obtained after the removal of DMSO and unreacted PEG or peptide. iRGD was conjugated on the surface of NPs via amidation reaction. The amine groups on iRGD reacted with carboxyl groups on NPs, which was activated by *N*‐(3‐(dimethylamino)propyl)‐*N*′‐ethylcarbodiimide hydrochloride (EDC) and sulfo‐*N*‐hydroxysuccinimide (Sulfo‐NHS). The IR780/Coumarin 6/Lexiscan/DOX/Ce6 ‐loaded NPs were fabricated based on the preparation method of empty NPs, except that polymer and IR780/coumarin 6/DOX/Ce6/Lexiscan were codissolved in DMSO. IR780/Coumarin 6 (dye/polymer = 0.5%, w/w) were loaded as in vivo imaging substance. Lexiscan (Lexiscan/polymer = 1%, w/w) was loaded as a BBB modulator agent. DOX and Ce6 were loaded as chemotherapy drug and sonosensitizer, respectively.

### Characterization of NE‐Responsiveness

The NE‐triggered responsive change of the NPs was analyzed using DLS and TEM. Typically, 1 mg mL^−1^ PEG‐PTD‐N‐PEG NPs were incubated with 100 nm NE in PBS at 37 °C. At predetermined time points (0, 10, and 60 min, 24 h), 100 µL of NP solutions were taken from the original system and diluted for hydrodynamic size testing with time. Changes in particle size and morphology were observed by TEM after 2% phosphotungstic acid negatively staining.

### Characterization of GSH Depletion In Vitro

The consumption of GSH was tested by UV–vis spectroscopy. 0.1 mg mL^−1^ PEG‐PTD‐N‐PEG NPs was mixed with 1 mm GSH at 37 °C. 50 µL of this solution was taken and added into 950 µL PBS at different time points. Then, 2 µL DTNB (10 mg mL^−1^) was added to above solution. After mixing by vortex, the absorbance spectrum of the solution was measured using multimode microplate reader (Spectramax M5, Molecular devices, USA).

### Quantitative Analysis of the Generation of ^1^O_2_

SOSG was employed to detect the production of ^1^O_2_ through the emission of green fluorescence. SOSG reagent (10 µm) was added into 3 mL Ce6‐loaded PEG‐PTD‐N‐PEG NPs (Ce6/polymer = 3%, w/w) in PBS. After different US irradiation (3 MHz, 1.5–3 W cm^−2^) durations, fluorescence measurements were made in a multimode microplate reader using excitation/emission of 488/525 nm.

### Tumor Inoculation

All animal work is compliant with relevant ethical regulations of the Yale University Institutional Animal Care and Utilization Committee (IACUC). For establishment of GL261 gliomas models, C57BL/6 mice (Charles River Laboratories, USA), ≈20 g each, were anesthetized through intraperitoneal injection of ketamine and xylazine. 5 × 10^4^ GL261 cells, which were engineered to express luciferase, were stereotactically injected into the right striatum using a stereotactic apparatus with an UltraMicroPump (UMP3) (World Precision Instruments, USA). Injection coordinates were 2 mm lateral and 0.5 mm posterior to the bregma at a depth of 3 mm from the cortical surface. The mice were then moved to a clean cage with free access to food and water, and monitored for the development of tumor by IVIS (Xenogen).

### Evaluation of the Therapeutic Benefits

Tumor‐bearing mice were randomly divided into 8 groups (*n* = 7 for each group) based on tumor sizes detected by IVIS 1 week after inoculation. Groups of mice received treatment of PBS, blank LiPTD NPs, free DOX, free DOX/Ce6, free DOX/Ce6 + US, DOX‐LiPTD NPs, DOX/Ce6‐LiPTD NPs, and DOX/Ce6‐LiPTD NPs + US, respectively. Treatments were conducted at day 8 after tumor inoculation and performed twice a week for 3 weeks. Tumor growth was monitored by IVIS Spectrum. The body weight change and survival were recorded. Four weeks after tumor inoculation, a cohort of mice were euthanized for pathological analysis. The brains were harvested and subjected to standard H&E and TUNEL staining. Mice with significant weight loss or neurological symptoms were humanely euthanized.

### Statistical Analysis

All data were collected in triplicate and presented as the means ± standard deviations. For comparison of different groups, unpaired, two‐tailed Student's *t*‐test was used. One‐way ANOVA was employed to compare the statistical significance of various treatments by GraphPad Prism 8.0 (*p < 0.05, **p < 0.01, ***p < 0.001, ****p < 0.0001).

## Conflict of Interest

The authors declare no conflict of interest.

## Supporting information

Supporting Information

## Data Availability

The data that support the findings of this study are available from the corresponding author upon reasonable request.
