# Supplementary material for: Targeted Delivery of Chemo‐Sonodynamic Therapy via Brain Targeting, Glutathione‐Consumable Polymeric Nanoparticles for Effective Brain Cancer Treatment
Source: Adv Sci (Weinh). 2022 Aug 15;9(28):2203894. doi: 10.1002/advs.202203894 (PMC9534955; doi:10.1002/advs.202203894)
Supplement: Supplementary file 1 — Supporting Information [file ADVS-9-2203894-s001.pdf]

Supporting Information

Targeted delivery of chemo-sonodynamic therapy via brain targeting, glutathione-consumable polymeric nanoparticles for effective brain cancer treatment

Haoan Wu, Xingchun Gao, Yuanyuan Luo, Jiang Yu, Gretchen Long, Zhaozhong Jiang, Jiangbing Zhou**

Materials**:** 3,3'-Dithiodipropionic acid (DTDP), 2,2'-thiodiethanol (TDE), MeO-PEG2K-OH, Evans blue were purchased from Sigma-Aldrich. Methoxy poly (ethylene glycol) maleimide [mPEG-MAL, molecular weight (MW): 5000] and maleimide poly (ethylene glycol) acetic acid (MAL-PEG-COOH, MW: 5000) were purchased from Jenkem Technology. NE-cleavable peptide (NH_2-RLQLKL-C-SH) and iRGD were purchased from AnaSpec. Chlorin e6, Lexiscan, and Doxorubicin (hydrochloride) were purchased from Cayman Chemical. All the chemicals were used as obtained without any further purification.

In vitro drug release**:** In vitro drug release was carried out using dialysis methods. DOX-loaded nanoparticles were suspended in a dialysis bag (molecular weight cutoff, 3 kDa) and dialyzed against 30 mL PBS (100 rpm, 37 °C) under different drug-release conditions. 0.5 mL of dialysis solution was collected and replaced with the fresh medium. In the collected fractions, the released DOX content was detected by a BioTek microplate reader at a wavelength of 254 nm according to the calibration curve of DOX. In the triggered release, 100 nM of NE or100 μM of GSH were uesd. To evaluate US-induced additional effects on drug release, DOX/Ce6-loaded NPs were dispersed in PBS and irradiated by US with intensity of 3 W/cm^2 for 3 min.

Cellular GSH depletion**:** For GSH detection, GL261 cells (1×10^5 cells/mL) were seeded into 6-well plates overnight and incubated with NPs at different concentrations for 24 h. Then the media was removed and the cells were stained with Thiol Tracker Violet (20 μM) for 30 min. Fluorescence microscope was used to observe the intracellular fluorescence.

Cellular ROS generation**:** To investigate intracellular ROS production, GL261 cells (1×10^5 cells/mL) were seeded into 6-well plates overnight and incubated with different NPs containing 5 μg/mL of Ce6 for 6 h. Then medium was removed and 20 μM DCFH-DA was added to cells for 20 min. Hereafter, US with 0.5 W/cm^2 intensity was applied to cells for 5 min. The intracellular DCF fluorescence of all groups was observed under fluorescence microscope (BZ-X800E).

***Fluorescent Imaging*:** Tumor bearing mice with comparable tumor loads were randomly assigned into experimental groups. IR780/coumarin 6-loaded NPs with the normalized fluorescence intensity were administered intravenously through the tail vein. 24h post-operation, brain and other main organs were isolated for IVIS imaging (Xenogen) with excitation wavelength of 745 nm and emission wavelength of 820. Then, the brains were fixed and sliced for fluorescence microscope imaging.

***Immunofluorescence staining*:** The brains in tumor-bearing mice were harvested and fixed in 4% paraformaldehyde. Then the brains were transferred into sucrose sloution with different concentration until the tissue sinks. Brain tissue were cut into 30 μm sections in thickness using a Leica CM1950 Cryostat and stained with Anti-Integrin α_V/β_3/CD51/CD61 Antibody (sc-7312, Santa Cruz Biotechnology). A647-conjugated Goat anti-Mouse IgG (A-21235, ThermoFisher Scientific) was used as secondary antibody. Images were captured using a All-in-One fluorescence microscope (BZ-X800E).

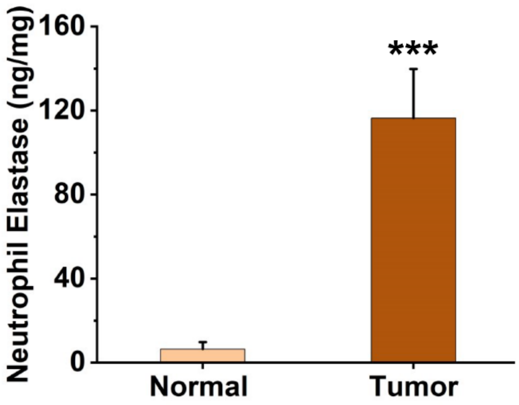

Figure S1. Quantification of NE in normal brain tissues and brain tumor.

(A)

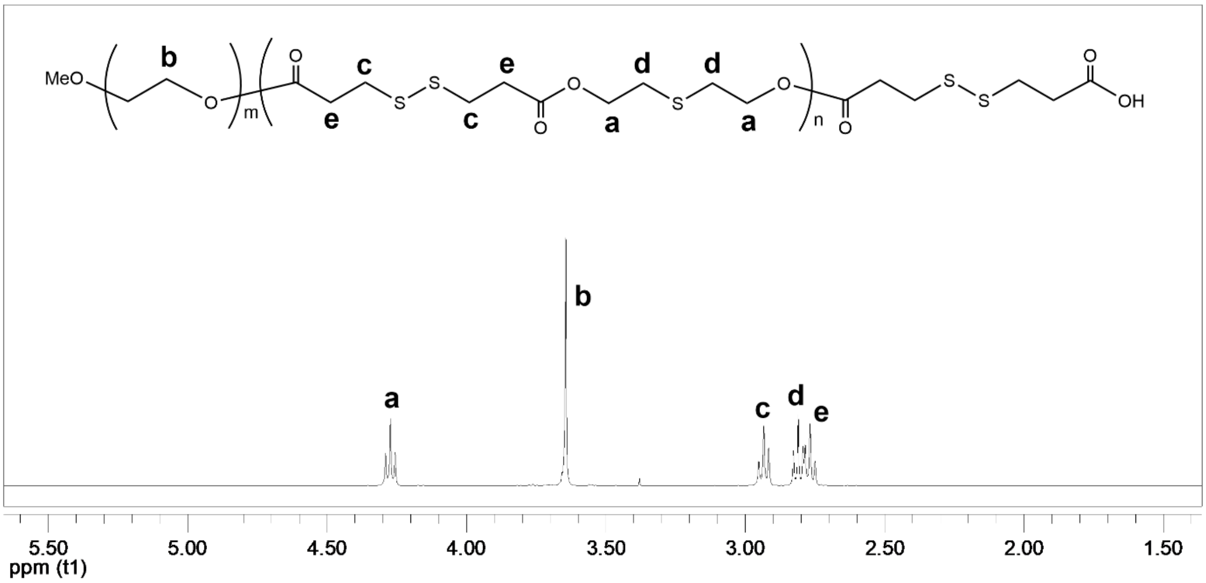

(B)

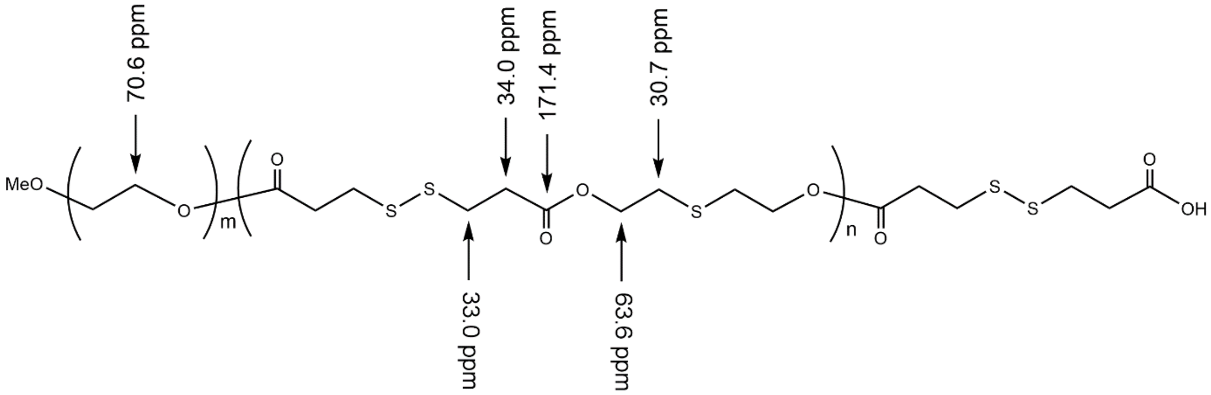

Figure S2. Structural assignments for (A) the proton and (B) carbon-13 NMR resonance absorptions of PEG-PTD block copolymer (solvent: chloroform-*d*).

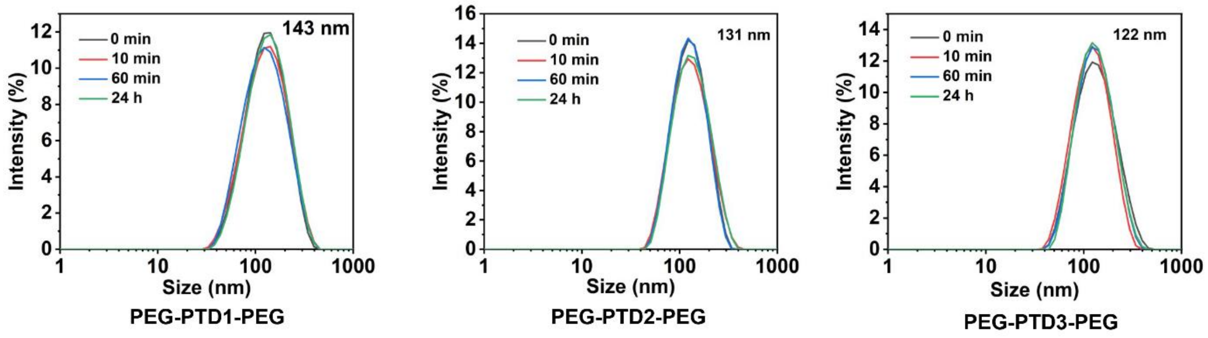

Figure S3. DLS changes of PEG-PTD-PEG NPs with and without NE.

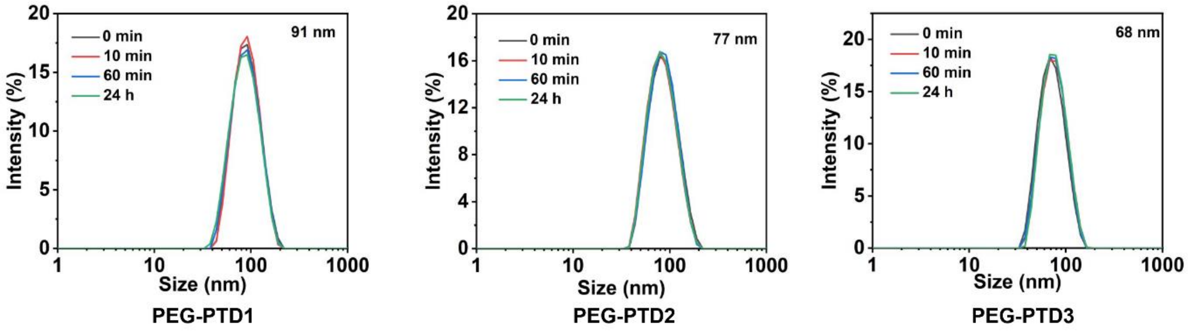

Figure S4. DLS changes of PEG-PTD NPs with and without NE.

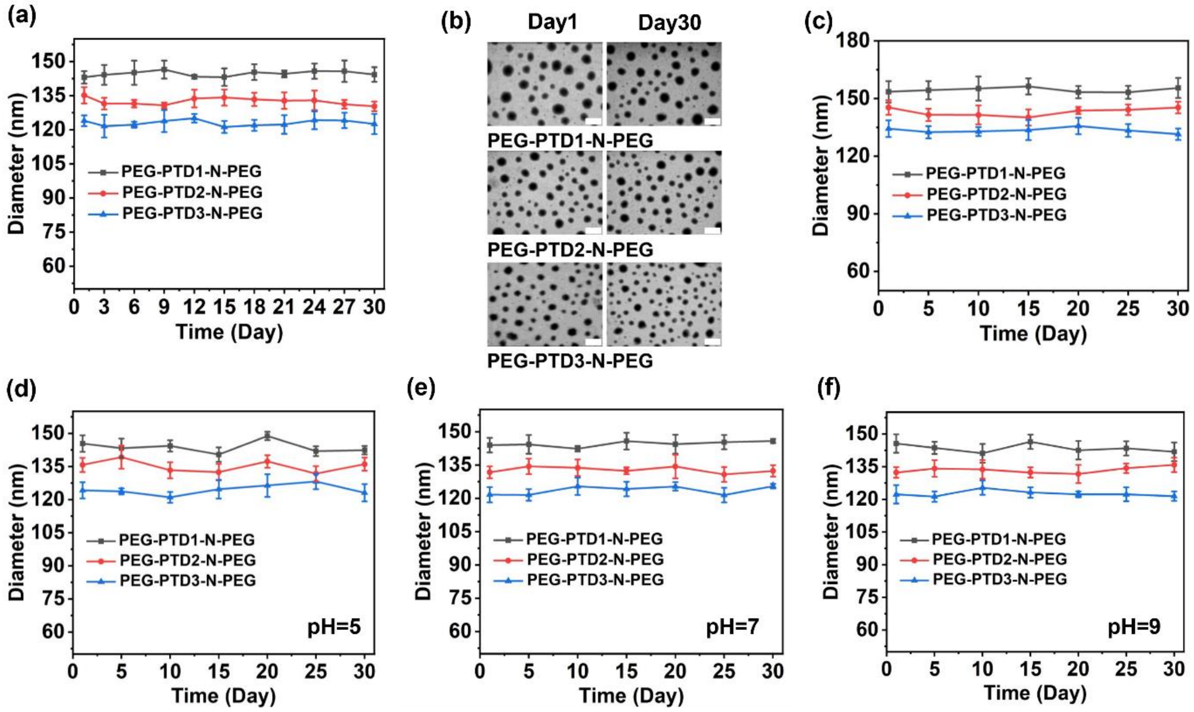

Figure S5. Characterization of NP stability. (a-b) DLS and TEM analyses of PEG-PTD-N-PEG NPs in PBS without NE for 30 days. (c) DLS analysis of serum stability of PEG-PTD-N-PEG NPs. (d-f) DLS analysis of PEG-PTD-N-PEG NPs in water with different pHs.

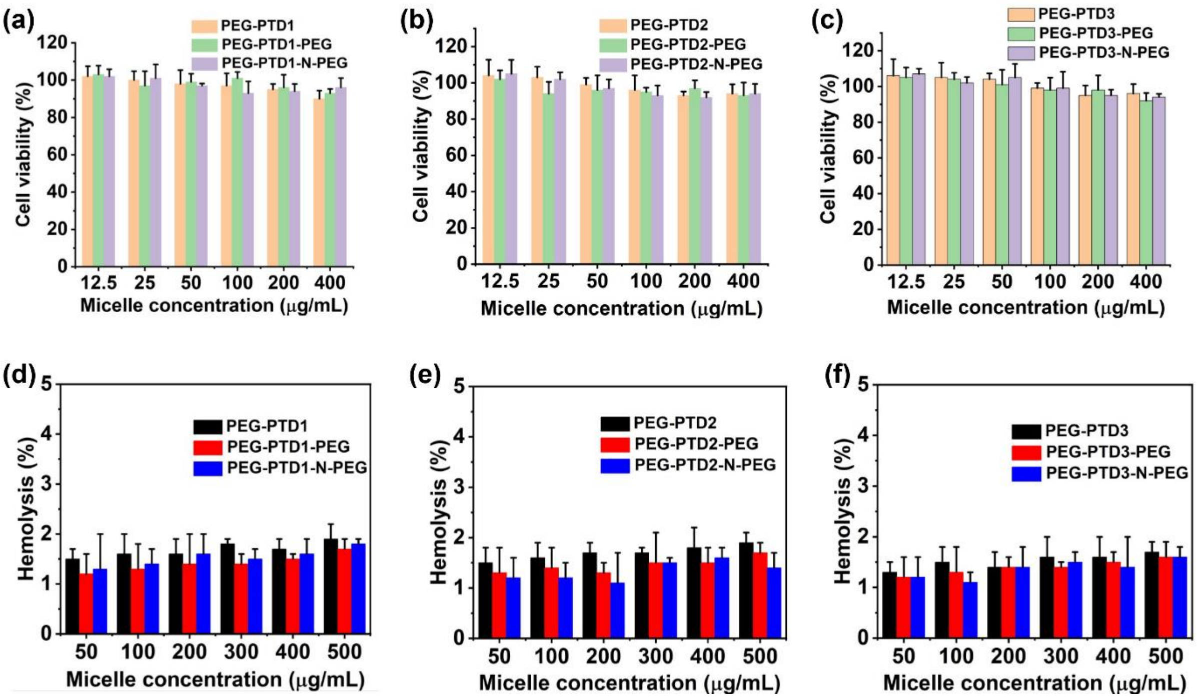

Figure S6. Characterization of the indicated NPs for their cytotoxicity by MTT assay (a-c) and blood compatibility by hemolysis assay (d-f).

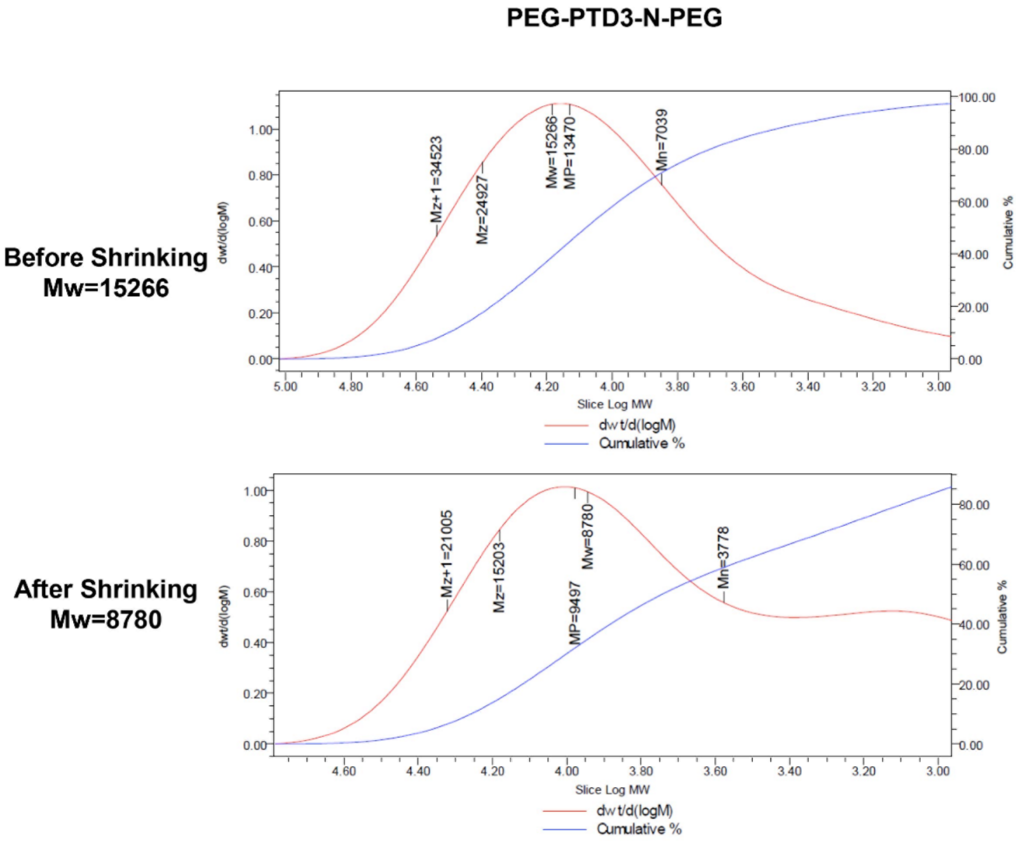

Figure S7. Characterization of the molecular weight of PEG-PTD3-N-PEG polymers before and after NP shrinkage by GPC.

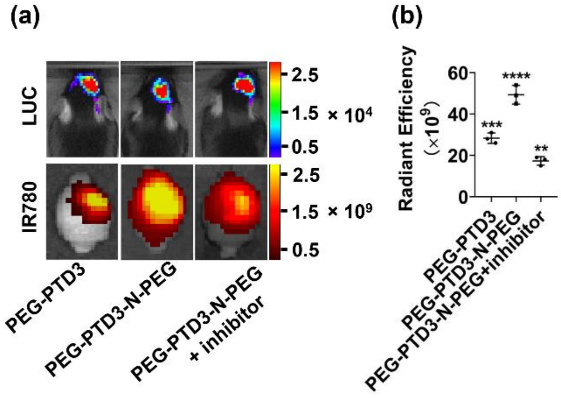

Figure S8. Characterization of PEG-PTD3-N-PEG NPs and PEG-PTD3 NPs for brain penetration. (a) Representative images and (b) quantification of tumor bearing mice receiving treatment of the indicated NPs.

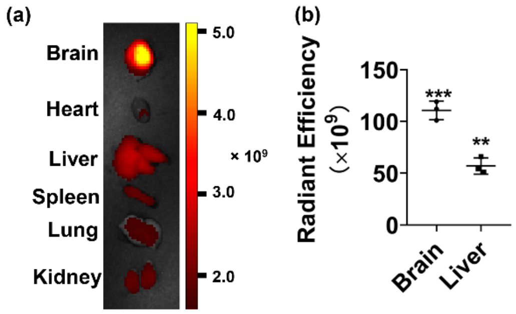

Figure S9. Ex vivo images (a) and quantification (b) of IR780-labeled LiPTD NPs in major organs.

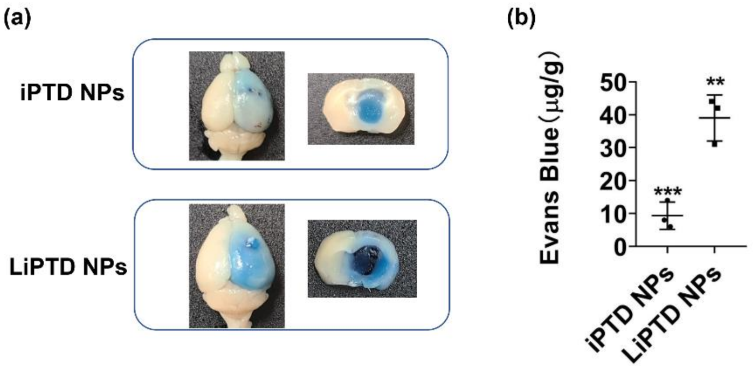

Figure S10. Representative images (a) and quantitfication (b) of Evans-blue leakage in the brain after the indicated treatment. Tumor-bearing mice received intravenous administration of iPTD NPs (LiPTD NPs without Lexiscan) and LiPTD NPs at 0, 24, and 48h. 2% Evans blue dye in PBS (5 mL/kg) was injected through the tail vein 24h after the last administration of NPs. After additional 12 hours, the brains were isolated and Evans blue dye was quantified.

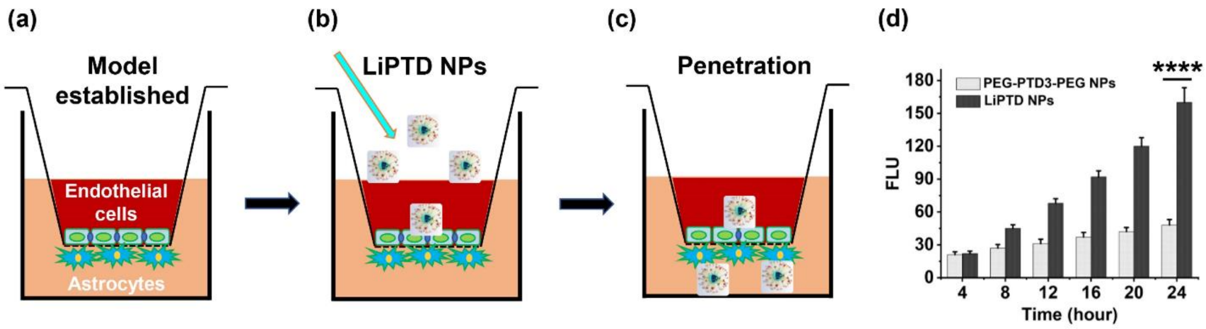

Figure S11. Characterization of BBB permeability of the indicated NPs. (a-c) Schematic diagram of the transwell-based BBB permeability assay. (d) Quantification of the permeability of Coumarin 6-loaded PEG-PTD3-PEG NPs and LiPTD NPs (50 μg/mL) at the indicated time points. Data represent mean ± S.D.; n = 3.

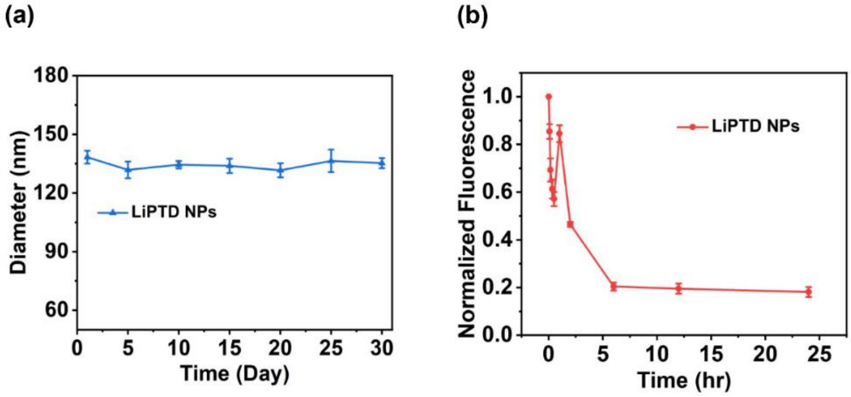

Figure S12. Characterization of serum stability (a) and blood circulation (b) of LiPTD NPs.

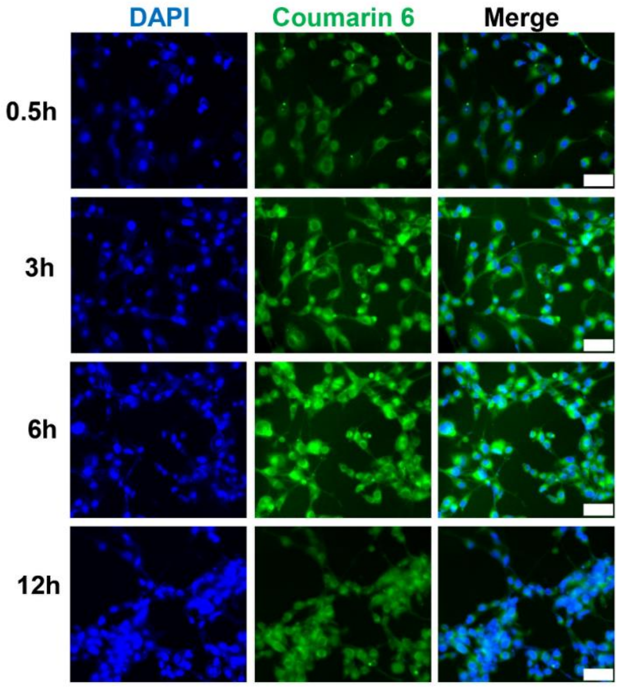

Figure S13. Representative images of GL261 cells after incubation with coumarin 6-loaded LiPTD NPs for the indicated time

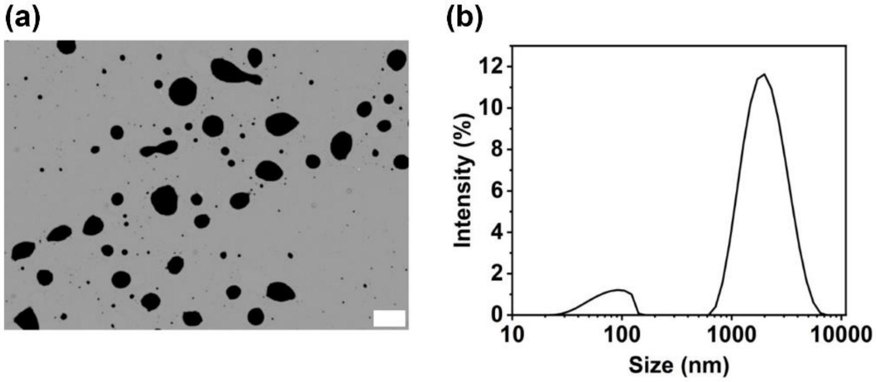

Figure S14. TEM image (a) and size distribution (b) of DOX/Ce6-loaded LiPTD NPs at 24h after US irradiation (3 W/cm^2, 5 min). Scare bar = 1 μm.

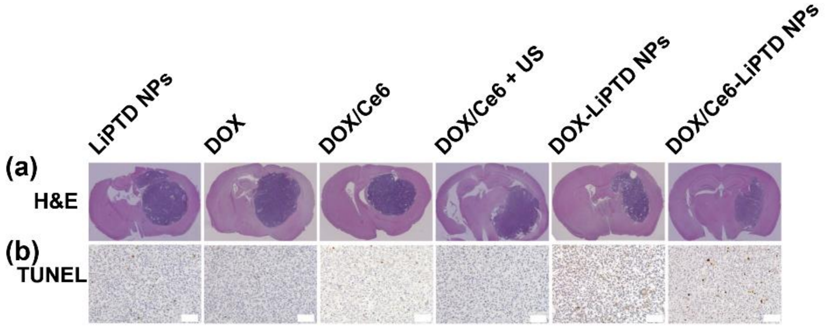

Figure S15. Representative images of immunohistochemical staining of brain tumors isolated from mice received the indicated treatments. Scale bar: 100 μm.

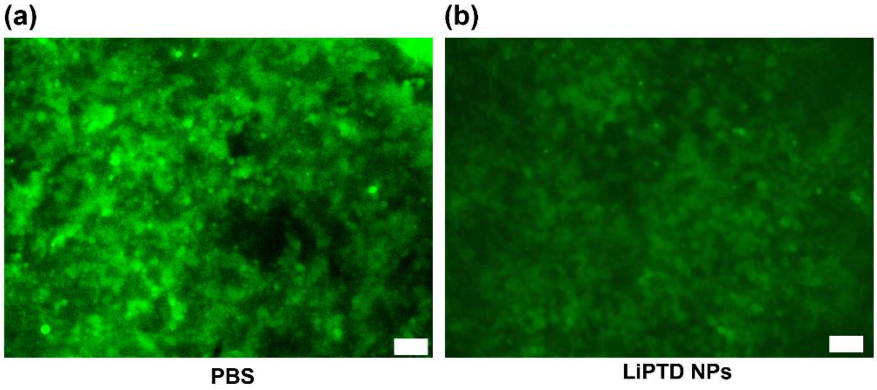

Figure S16. Fluorescence images of tumor slices with indicated treatments after Thiol Tracker violet staining. Scare bar = 50 μm.

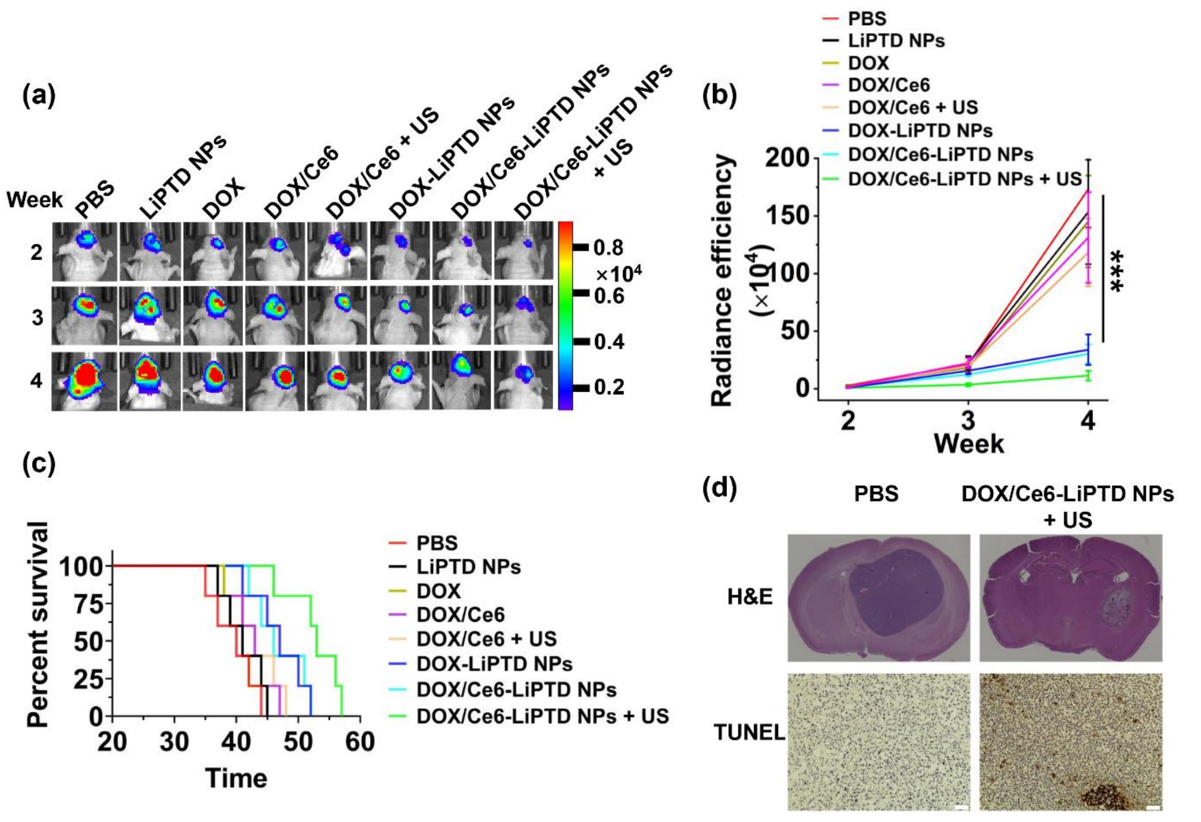

Figure S17. Evaluation of DOX/Ce6-loaded LiPTD NPs for treatment of U87 gliomas. (a) Representative images and (b) semi-quantification of luciferase signals in brain tumors in mice received the indicated treatments. (c) Kaplan–Meier survival analysis of mice received the indicated treatments (n = 5). (d) Representative immunohistochemical staining of brain tumors isolated from mice received the indicated treatments. Scale bar: 50 μm.

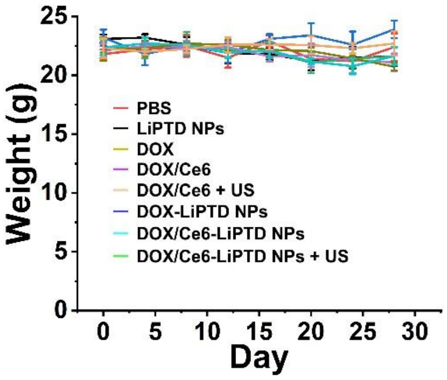

Figure S18. Body weight analysis of GL261 brain tumor-bearing mice with the indicated treatments.

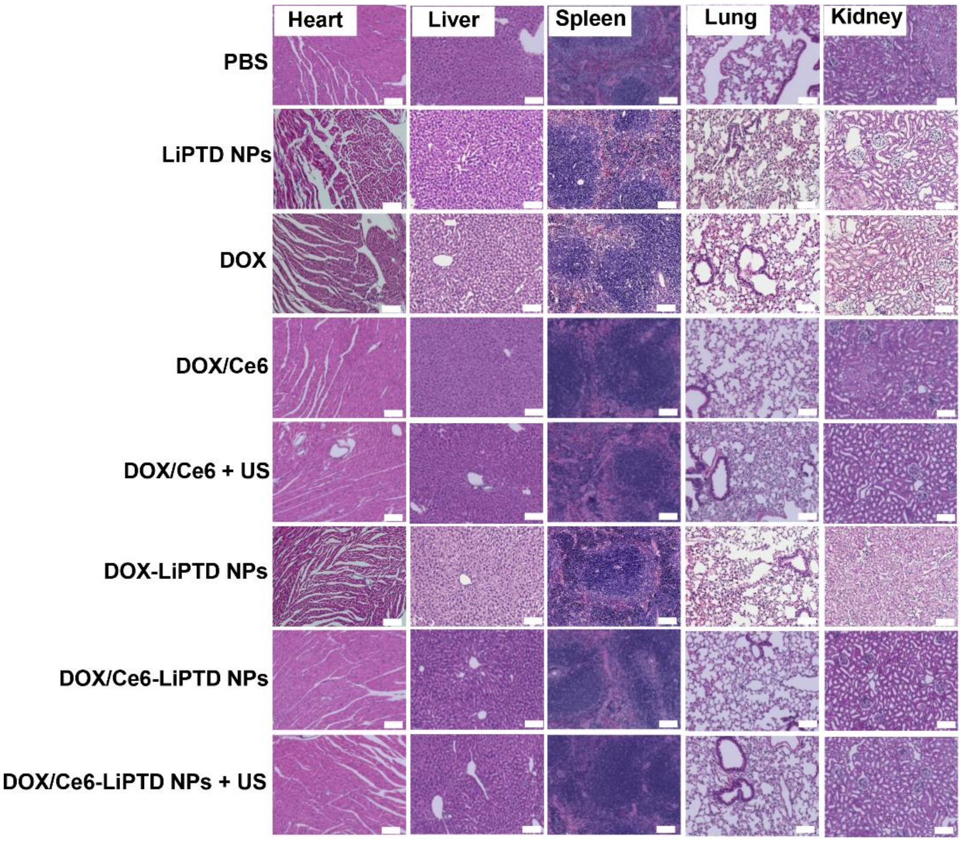

Figure S19. Representative images of H&E staining for major organs isolated from mice received the indicated treatments.

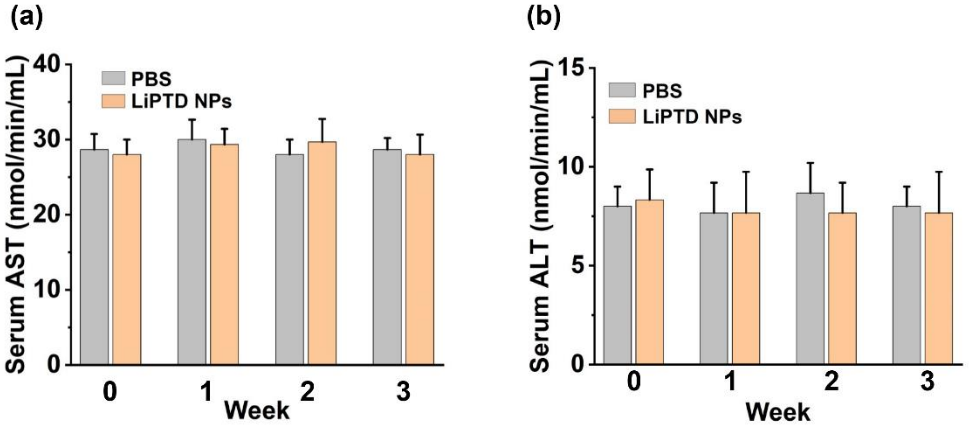

Figure S20. Quantification of serum AST and ALT levels with time in mice received the indicated treatments.

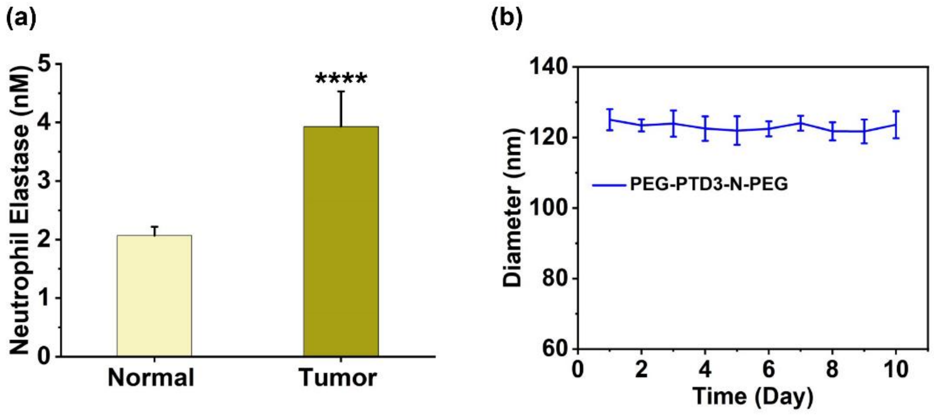

Figure S21. Characterization of NE elevation in the blood and stability of PEG-PTD-N-PEG NPs. (a) Quantification of the concentration of NE in the blood in tumor mice. (b) Change of the size of PEG-PTD3-N-PEG NPs with time after incubation in PBS buffer with 3.9 nM NE.

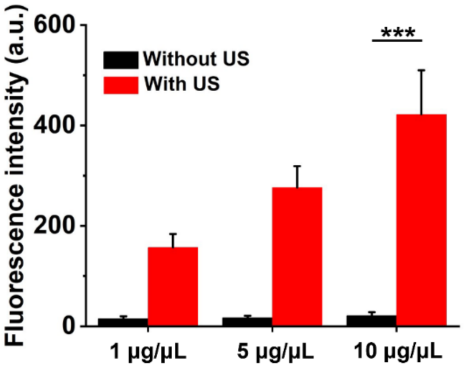

Figure S22. Quantification of singlet oxygen production in tumors. Tumor-bearing mice were randomly divided into 2 groups (n = 3), which received intratumorally administration of 5μL Ce6-loaded LiPTD NPs (Ce6/polymer = 3%, w/w) with SOSG reagent (20 μM). Ten minutes after injection, US irradiation (3 MHz, 3 W/cm^2, 10 min) was performed on the skull of mice. After 5 min, the mice were euthanized. Tumor tissues were isolated and homogenized in 100 μL of PBS. Fluorescence measurements were made in a multi-mode microplate reader using excitation/emission of 488/525 nm.
